# 2,2′-[(2S*,6R*)-Piperidine-2,6-di­yl]­di­pro­pan-2-ol

**DOI:** 10.1107/S1600536812005879

**Published:** 2012-02-24

**Authors:** Guillaume Journot, Reinhard Neier, Helen Stoeckli-Evans

**Affiliations:** a Institute of Chemistry, University of Neuchâtel, Avenue de Bellevaux 51, CH-2000 Neuchâtel, Switzerland; b Institute of Physics, University of Neuchâtel, Rue Emile-Argand 11, CH-2000 Neuchâtel, Switzerland

## Abstract

In the title compound, C_11_H_23_NO_2_, the piperidine ring has a chair conformation. The two hy­droxy H atoms are disordered over two positions with fixed occupancy ratios of 0.57:0.43 and 0.63:0.37. In the mol­ecule, there are two short N—H⋯O inter­actions. In the crystal, four symmetry-related mol­ecules are linked by O—H⋯O hydrogen bonds to form a cage-like arrangement, centered about the point of inter­section of three twofold axes. These cages stack along the [100] direction.

## Related literature

For literature on ligands of the pincer-type family, see: van Koten (1989 ▶); Albrecht & van Koten (2001 ▶). For metal complexes of such pincer ligands, see: Hofmeier & Schubert (2004 ▶); Li *et al.* (2007 ▶). For the synthesis of the starting material 2,2′-(pyridine-2,6-di­yl)dipropan-2-ol, see: Klein *et al.* (2009 ▶). For an example of the transformation of bis-benzylic alcohols of 2,6-disubstituted pyridines, see: Klein *et al.* (2009 ▶). For the crystal structure of *cis*-(piperidine-2,6-di­yl)di­me­than­ol, see: Hartung *et al.* (2007 ▶).
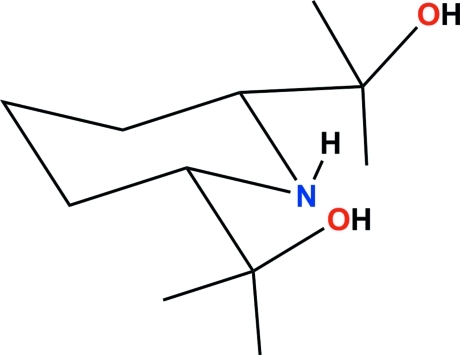


## Experimental

### 

#### Crystal data

C_11_H_23_NO_2_
*M* *_r_* = 201.30
Orthorhombic, 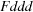

*a* = 12.0713 (9) Å
*b* = 23.4762 (10) Å
*c* = 34.496 (2) Å
*V* = 9775.8 (10) Å^3^
*Z* = 32
Mo *K*α radiation
μ = 0.07 mm^−1^
*T* = 173 K
0.45 × 0.45 × 0.40 mm

#### Data collection

Stoe IPDS 2 diffractometer
Absorption correction: multi-scan (*MULscanABS* in *PLATON*; Spek, 2009 ▶) *T*_min_ = 0.911, *T*_max_ = 1.000
32226 measured reflections
2319 independent reflections
1499 reflections with *I* > 2σ(*I*)
*R*_int_ = 0.135

#### Refinement

*R*[*F* ^2^ > 2σ(*F* ^2^)] = 0.086
*wR*(*F* ^2^) = 0.146
*S* = 1.16
2319 reflections
135 parameters
4 restraints
H atoms treated by a mixture of independent and constrained refinement
Δρ_max_ = 0.16 e Å^−3^
Δρ_min_ = −0.15 e Å^−3^

### 

Data collection: *X-AREA* (Stoe & Cie, 2009 ▶); cell refinement: *X-AREA*; data reduction: *X-RED32* (Stoe & Cie, 2009 ▶); program(s) used to solve structure: *SHELXS97* (Sheldrick, 2008 ▶); program(s) used to refine structure: *SHELXL97* (Sheldrick, 2008 ▶); molecular graphics: *PLATON* (Spek, 2009 ▶) and *Mercury* (Macrae *et al.*, 2008 ▶); software used to prepare material for publication: *SHELXL97*, *PLATON* and *publCIF* (Westrip, 2010 ▶).

## Supplementary Material

Crystal structure: contains datablock(s) I, global. DOI: 10.1107/S1600536812005879/pk2389sup1.cif

Structure factors: contains datablock(s) I. DOI: 10.1107/S1600536812005879/pk2389Isup2.hkl

Supplementary material file. DOI: 10.1107/S1600536812005879/pk2389Isup3.cml

Additional supplementary materials:  crystallographic information; 3D view; checkCIF report

## Figures and Tables

**Table 1:** Hydrogen-bond geometry (Å, °)

*D*—H⋯*A*	*D*—H	H⋯*A*	*D*⋯*A*	*D*—H⋯*A*
N1—H1⋯O1′	0.84 (3)	2.38 (3)	2.792 (3)	111 (2)
N1—H1⋯O1′′	0.84 (3)	2.43 (3)	2.814 (3)	109 (2)
O1′—H1*A*⋯O1′′^i^	0.82	1.99	2.805 (3)	169
O1′—H1*B*⋯O1′^ii^	0.83	1.99	2.807 (4)	167
O1′′—H1*C*⋯O1′^i^	0.82	2.00	2.805 (3)	171
O1′′—H1*D*⋯O1′′^iii^	0.83	2.03	2.762 (5)	148

Symmetry codes: (i) 
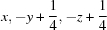
; (ii) 
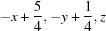
; (iii) 
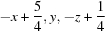
.

**Fig. 1.**
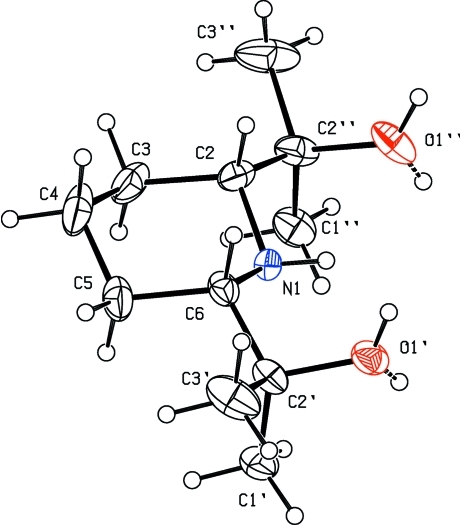
A view of the molecular structure of the title molecule (2), with displacement ellipsoids drawn at the 30% probability level. (The O···H dashed lines indicate the positions of the minor components of the hydroxyl H atoms.)

**Fig. 2.**
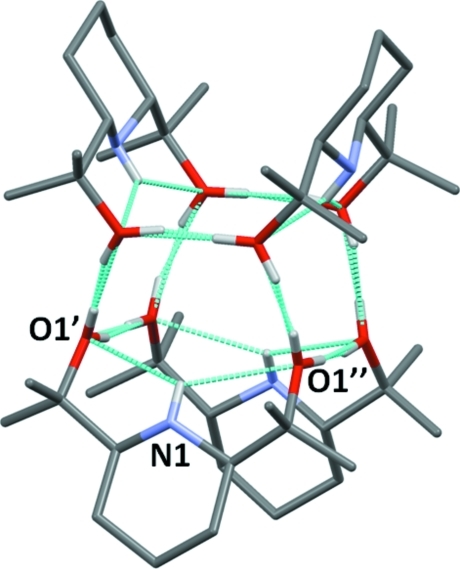
A view of the hydrogen bonded cage formed by four symmety related molecules of the title compound. The C-bound H atoms have beem omitted for clarity. The O—H···O and N—H···O hydrogen bonds are shown as dashed cyan lines (see Table 1 for details).

**Fig. 3.**
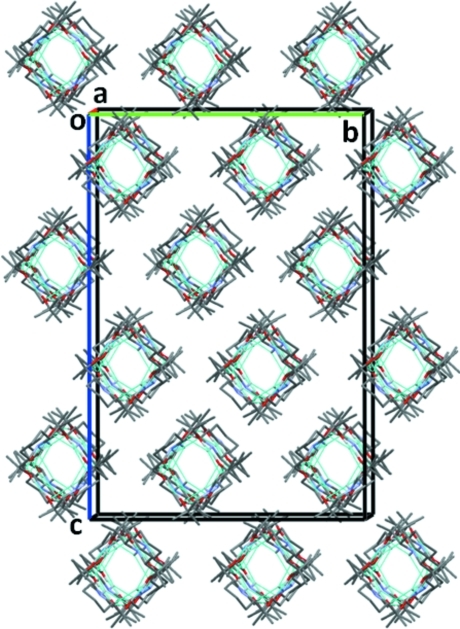
A view along the a axis of the crystal packing of the title compound. The C-bound H atoms have beem omitted for clarity. The O—H···O and N—H···O hydrogen bonds are shown as dashed cyan lines (see Table 1 for details).

**Fig. 4.**
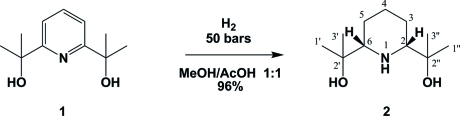
Reaction scheme for the synthesis of the title compound, (2).

## supplementary crystallographic information

### Comment

Terpyridine and its derivatives are prototypical ligands of the pincer type family (Van Koten, 1989; Albrecht & van Koten, 2001). They have been widely used in coordination chemistry (Hofmeier & Schubert, 2004). The metal complexes obtained from pincer ligands are conformationally restricted and often thermodynamically highly stable (Hofmeier & Schubert, 2004; Li *et al.*, 2007). The bis-benzylic alcohols of 2,6-disubstituted pyridines belonging to this class of ligands can be easily transformed (Klein *et al.*, 2009).

The modification of these ligands by the hydrogenation of the pyridine ring installs chirality into the structure and increases the basicity and the strength of the ligand. In contrast to the 2,6-pyridinedicarboxylic acids and its derivatives, which have been extensively used, studies on the corresponding tridentate ONO-piperidine ligands containing the bis-alcohols have been very rare so far. The title compound (2) was prepared by the stereoselective *cis*-reduction of 2,2'-(pyridine-2,6-diyl)dipropan-2-ol (1). Herein we report on the synthesis and the crystal structure of the title compound, (2).

The molecular structure of the title molecule is illustrated in Fig. 1. The geometric parameters are very similar to those found for *cis*-(piperidine-2,6-diyl)dimethanol (Hartung *et al.*, 2007). The piperidine ring has a chair conformation, with atoms N1 and C4 being displaced from the plane through atoms C2/C3/C5/C6 by 0.667 (2) and -0.662 (3) Å, respectively.

In the molecule the amine (N1) H atom is involved in two short interactions with the hydroxyl O atoms, O1' and O1'' (Table 1). The hydroxyl H atoms are each disordered over two positions, H1A/H1B and H1C/H1D. Their occupancies were initially refined before being fixed at 0.57/0.43 and 0.63/0.37, respectively. The ^1^H NMR signal for the hydroxyl H atoms [δ 2.88 (bs, 2 H, OH); see archived CIF] is a broad singlet, which indicates some fluxionality of these protons in solution.

In the crystal, four symmetry related molecules are linked by O—H···O hydrogen bonds to form a cage-like arrangement, centered about the point of intersection of three 2-fold axes (Fig 2). These cages are arranged in stacks along direction [100], as shown in Fig. 3.

### Experimental

The synthesis of the title compound (2) is illustated in Fig. 4. The starting material, 2,2'-(pyridine-2,6-diyl)dipropan-2-ol (1), was prepared in one step from the commercially available dimethyl pyridine-2,6-dicarboxylate, according to the method described by (Klein *et al.*, 2009). The title compound (2), was synthesized by heating 0.5 g (2.56 mmol) of compound (1), together with 10% Pd/C (430 mg), methanol (10 ml) and acetic acid (10 ml), in an autoclave under hydrogen (50 atm), with stirring at 323 K for 12 h. For workup the reaction was filtered through a pad of celite and washed three times with dichloromethane. The solution was concentrated under vacuum to give a colourless slurry. The slurry was dissolved in dichloromethane and washed with 5% sodium hydroxide and the mixture was stirred for 5 min. The organic layer was separated and the aqueous layer was extracted three times with dichloromethane. The combined organic layers were washed with brine, dried with sodium sulfate and concentrated under vacuum to yield 0.494 g (96%) of compound (2). Melting point: 345.3 K. HRMS calcd. for [C_11_H_23_NO_2_^+^H^+^] 224.1621; found 224.1621. Colourless rod-like crystals were obtained by slow evaporation of a solution of (2) in dichloromethane. Spectroscopic data for the title compound (2), are given the archived CIF.

### Refinement

The NH H-atom was located in a difference Fourier map and was freely refined. The OH H atoms are disordered over two positions. They were located in a difference Fourier map and were initially freely refined, including their occupancies, before being refined with distance restraints of 0.84 (2) Å. In the final cycles of refinement they were refined with fixed occupancies of 0.57/0.43 and 0.63/0.37, and allowed to ride on the parent O atom with *U*_iso_(H) = 1.5*U*_eq_(O). The C-bound H-atoms were included in calculated positions and treated as riding atoms: C—H = 0.98, 0.99 and 1.00 Å for CH_3_, CH_2_ and CH H-atoms, respectively, with *U*_iso_(H) = k × *U*_eq_(parent C-atom), where k = 1.5 for CH_3_ H-atoms and k = 1.2 for all other H-atoms.

### Crystal data

**Table:**

C_11_H_23_NO_2_	*D*_x_ = 1.094 Mg m^−^^3^
*M**_r_* = 201.30	Melting point: 345.3 K
Orthorhombic, *F**d**d**d*	Mo *K*α radiation, λ = 0.71073 Å
Hall symbol: -F 2uv 2vw	Cell parameters from 13139 reflections
*a* = 12.0713 (9) Å	θ = 2.0–24.3°
*b* = 23.4762 (10) Å	µ = 0.07 mm^−^^1^
*c* = 34.496 (2) Å	*T* = 173 K
*V* = 9775.8 (10) Å^3^	Rod, colourless
*Z* = 32	0.45 × 0.45 × 0.40 mm
*F*(000) = 3584	

### Data collection

**Table:**

Stoe IPDS 2 diffractometer	2319 independent reflections
Radiation source: fine-focus sealed tube	1499 reflections with *I* > 2σ(*I*)
Graphite monochromator	*R*_int_ = 0.135
φ & ω scans	θ_max_ = 25.7°, θ_min_ = 2.0°
Absorption correction: multi-scan (MULscanABS in *PLATON*; Spek, 2009)	*h* = −14→14
*T*_min_ = 0.911, *T*_max_ = 1.000	*k* = −28→28
32226 measured reflections	*l* = −42→41

### Refinement

**Table:**

Refinement on *F*^2^	Primary atom site location: structure-invariant direct methods
Least-squares matrix: full	Secondary atom site location: difference Fourier map
*R*[*F*^2^ > 2σ(*F*^2^)] = 0.086	Hydrogen site location: inferred from neighbouring sites
*wR*(*F*^2^) = 0.146	H atoms treated by a mixture of independent and constrained refinement
*S* = 1.16	*w* = 1/[σ^2^(*F*_o_^2^) + (0.0468*P*)^2^ + 7.437*P*] where *P* = (*F*_o_^2^ + 2*F*_c_^2^)/3
2319 reflections	(Δ/σ)_max_ < 0.001
135 parameters	Δρ_max_ = 0.16 e Å^−^^3^
4 restraints	Δρ_min_ = −0.15 e Å^−^^3^

### Special details

**Table:**

Experimental. Spectroscopic data for 2,2'-((2*S**,6*R**)-piperidine-2,6-diyl)dipropan-2-ol (2):^1^H NMR (CDCl_3_, 298 K, p.p.m.) δ 2.88 (bs, 2 H, OH), 2.47 (d, ^3^J(2, 3 b) and ^3^J(6, 5 b) = 11.4 Hz, 2 H, C—H(2, 6)), 1.97 (dquint, ^3^J(4 b, 4a) = 13.3 Hz, ^3^J(4 b, 3 b) = 3 J(4 b, 5 b) = 3.3 Hz, ^3^J(4 b, 3a) = ^3^J(4 b, 5a) = 3.3 Hz, 1 H, C—H(4 b)), 1.74 (dd, ^3^J(3a, 3 b) = 12.8 Hz and ^3^J(5a, 5 b) = 12.8 Hz, ^3^J(3a, 4 b) = 3.0 Hz and ^3^J(5a, 4 b) = 3.0 Hz, 2 H, C—H(3a, 5a)), 1.45 (qt, ^3^J(4a, 4 b) = 13.0 Hz, ^3^J(4a, 3 b) = 13.0 Hz and ^3^J(4a, 5 b) = 13.0 Hz, 3 J(4a, 3a) = 3.7 Hz and ^3^J(4a, 5a) = 3.7 Hz, 1 H, C—H(4a)), 1.25 (s, 6 H, CH_3_), 1.16 (s, 6 H, CH_3_), 1.06 (qd, ^3^J(3 b, 2) = 12.3 Hz and ^3^J(5 b, 6) = 12.3 Hz, ^3^J(3 b, 3a) = 12.3 Hz and ^3^J(5 b, 5a) = 12.3 Hz, ^3^J(3 b, 4a) = 12.3 Hz and ^3^J(5 b, 4a) = 12.3 Hz, ^3^J(3 b, 4 b) = 3.3 Hz and ^3^J(5 b, 4 b) = 3.3 Hz, 2 H, CH_2_(3 b, 5 b));^13^C NMR (CDCl_3_, 298 K, p.p.m.) δ 71.67 (C(2, 2')), 65.45 (C(2, 6)), 27.45 (CH_3_), 26.91 (C(3, 5)), 24.69 (C(4)), 24.36 (CH_3_).IR (KBr, cm^-1^): 3377 b s, 2982 s, 2944 s, 2855*m*, 2782*m*, 2694w, 2586w, 1456*m*, 1442*m*, 1380 s, 1130 s, 931 s, 822 s, 537w.
Geometry. Bond distances, angles *etc*. have been calculated using the rounded fractional coordinates. All su's are estimated from the variances of the (full) variance-covariance matrix. The cell e.s.d.'s are taken into account in the estimation of distances, angles and torsion angles
Refinement. The NH H-atom was located in a difference Fourier map and was freely refined. The OH H atoms are disordered over two positions with fixed occupancies of 0.57/0.43 and 0.63/0.37. They were located in a difference Fourier map and were initially freely refined, including their occupancies, before being refined with distance restraints of 0.84 (2) Å. In the final cycles of refinement they were refined with fixed occupancies of 0.57/0.43 and 0.63/0.37, and allowed to ride on the parent O atom with *U*_iso_(H) = 1.5*U*_eq_(O). The C-bound H-atoms were included in calculated positions and treated as riding atoms: C—H = 0.98, 0.99 and 1.00 Å for CH_3_, CH_2_ and CH H-atoms, respectively, with *U*_iso_(H) = k × *U*_eq_(parent C-atom), where k = 1.5 for CH_3_ H-atoms and k = 1.2 for all other H-atoms.

### Fractional atomic coordinates and isotropic or equivalent isotropic displacement parameters (Å^2^)

**Table:**

	*x*	*y*	*z*	*U*_iso_*/*U*_eq_	Occ. (<1)
O1'	0.52422 (16)	0.09518 (8)	0.04534 (5)	0.0474 (7)	
O1''	0.51927 (19)	0.22777 (8)	0.14029 (6)	0.0602 (8)	
N1	0.41660 (19)	0.19356 (10)	0.07083 (6)	0.0344 (7)	
C1'	0.4778 (3)	0.15489 (12)	−0.00855 (8)	0.0490 (10)	
C1''	0.4860 (3)	0.30648 (12)	0.09761 (9)	0.0551 (11)	
C2	0.3546 (2)	0.22205 (11)	0.10141 (8)	0.0426 (10)	
C2'	0.4316 (2)	0.11656 (11)	0.02325 (8)	0.0410 (10)	
C2''	0.4314 (3)	0.26220 (11)	0.12374 (8)	0.0496 (10)	
C3	0.2533 (3)	0.25074 (14)	0.08304 (10)	0.0602 (13)	
C3'	0.3740 (3)	0.06505 (14)	0.00542 (10)	0.0730 (14)	
C3''	0.3697 (4)	0.29130 (15)	0.15692 (10)	0.0900 (16)	
C4	0.1844 (2)	0.20695 (15)	0.06117 (11)	0.0712 (13)	
C5	0.2544 (2)	0.17559 (14)	0.03176 (10)	0.0554 (11)	
C6	0.3549 (2)	0.14843 (11)	0.05111 (8)	0.0391 (9)	
H1	0.472 (2)	0.1777 (11)	0.0809 (7)	0.035 (8)*	
H1A	0.51390	0.07430	0.06420	0.0710*	0.570
H1C	0.51740	0.20950	0.16040	0.0900*	0.630
H1L	0.51510	0.18770	0.00320	0.0740*	
H1M	0.41710	0.16820	−0.02510	0.0740*	
H1N	0.53100	0.13340	−0.02420	0.0740*	
H1O	0.53320	0.28730	0.07860	0.0830*	
H1P	0.53110	0.33230	0.11340	0.0830*	
H1Q	0.42860	0.32830	0.08410	0.0830*	
H2	0.32750	0.19230	0.11990	0.0510*	
H3A	0.20760	0.26860	0.10350	0.0720*	
H3B	0.27790	0.28100	0.06500	0.0720*	
H3C	0.42710	0.04350	−0.01030	0.1090*	
H3D	0.31270	0.07790	−0.01100	0.1090*	
H3E	0.34510	0.04060	0.02610	0.1090*	
H3F	0.32950	0.26260	0.17200	0.1350*	
H3G	0.31710	0.31900	0.14630	0.1350*	
H3H	0.42300	0.31090	0.17370	0.1350*	
H4A	0.15250	0.17930	0.07980	0.0850*	
H4B	0.12240	0.22640	0.04780	0.0850*	
H5A	0.20940	0.14570	0.01910	0.0670*	
H5B	0.27950	0.20250	0.01150	0.0670*	
H6	0.32790	0.12090	0.07110	0.0470*	
H1B	0.57870	0.11630	0.04260	0.0710*	0.430
H1D	0.57710	0.24160	0.13160	0.0900*	0.370

### Atomic displacement parameters (Å^2^)

**Table:**

	*U*^11^	*U*^22^	*U*^33^	*U*^12^	*U*^13^	*U*^23^
O1'	0.0614 (13)	0.0440 (11)	0.0367 (12)	0.0143 (10)	−0.0002 (10)	0.0061 (9)
O1''	0.0968 (18)	0.0448 (12)	0.0391 (12)	0.0016 (12)	−0.0205 (12)	0.0086 (10)
N1	0.0292 (12)	0.0401 (13)	0.0340 (13)	0.0063 (11)	−0.0009 (11)	0.0029 (11)
C1'	0.0569 (19)	0.0521 (18)	0.0381 (17)	0.0011 (15)	0.0021 (15)	0.0106 (14)
C1''	0.073 (2)	0.0422 (17)	0.050 (2)	−0.0020 (16)	−0.0034 (17)	0.0073 (15)
C2	0.0459 (17)	0.0385 (15)	0.0435 (18)	0.0099 (14)	0.0160 (14)	0.0092 (14)
C2'	0.0513 (18)	0.0373 (16)	0.0345 (16)	−0.0044 (14)	−0.0085 (14)	0.0051 (13)
C2''	0.076 (2)	0.0388 (15)	0.0340 (17)	0.0091 (16)	0.0109 (16)	0.0048 (14)
C3	0.0441 (18)	0.0564 (19)	0.080 (3)	0.0191 (15)	0.0206 (17)	0.0162 (19)
C3'	0.112 (3)	0.057 (2)	0.050 (2)	−0.026 (2)	−0.014 (2)	−0.0008 (17)
C3''	0.158 (4)	0.061 (2)	0.051 (2)	0.023 (2)	0.038 (3)	−0.0051 (19)
C4	0.0315 (17)	0.075 (2)	0.107 (3)	0.0104 (17)	−0.0006 (19)	0.037 (2)
C5	0.0342 (17)	0.066 (2)	0.066 (2)	−0.0054 (15)	−0.0126 (16)	0.0203 (18)
C6	0.0359 (15)	0.0429 (16)	0.0384 (17)	−0.0065 (13)	−0.0060 (13)	0.0128 (13)

### Geometric parameters (Å, º)

**Table:**

O1'—C2'	1.443 (3)	C1'—H1M	0.9800
O1''—C2''	1.451 (4)	C1'—H1N	0.9800
O1'—H1B	0.8300	C1''—H1O	0.9800
O1'—H1A	0.8200	C1''—H1P	0.9800
O1''—H1C	0.8200	C1''—H1Q	0.9800
O1''—H1D	0.8300	C2—H2	1.0000
N1—C6	1.463 (3)	C3—H3A	0.9900
N1—C2	1.456 (3)	C3—H3B	0.9900
N1—H1	0.84 (2)	C3'—H3C	0.9800
C1'—C2'	1.525 (4)	C3'—H3D	0.9800
C1''—C2''	1.526 (4)	C3'—H3E	0.9800
C2—C2''	1.530 (4)	C3''—H3F	0.9800
C2—C3	1.533 (4)	C3''—H3G	0.9800
C2'—C3'	1.525 (4)	C3''—H3H	0.9800
C2'—C6	1.530 (4)	C4—H4A	0.9900
C2''—C3''	1.527 (5)	C4—H4B	0.9900
C3—C4	1.522 (5)	C5—H5A	0.9900
C4—C5	1.512 (5)	C5—H5B	0.9900
C5—C6	1.524 (4)	C6—H6	1.0000
C1'—H1L	0.9800		
			
C2'—O1'—H1A	120.00	H1O—C1''—H1P	109.00
C2'—O1'—H1B	110.00	H1O—C1''—H1Q	109.00
C2''—O1''—H1C	127.00	H1P—C1''—H1Q	110.00
C2''—O1''—H1D	105.00	N1—C2—H2	108.00
C2—N1—C6	114.1 (2)	C2''—C2—H2	108.00
C6—N1—H1	106.1 (18)	C3—C2—H2	108.00
C2—N1—H1	108.2 (17)	C2—C3—H3A	110.00
N1—C2—C3	108.2 (2)	C2—C3—H3B	110.00
N1—C2—C2''	109.7 (2)	C4—C3—H3A	110.00
C2''—C2—C3	114.9 (2)	C4—C3—H3B	110.00
O1'—C2'—C6	107.9 (2)	H3A—C3—H3B	108.00
O1'—C2'—C1'	107.6 (2)	C2'—C3'—H3C	110.00
O1'—C2'—C3'	106.9 (2)	C2'—C3'—H3D	109.00
C1'—C2'—C6	112.6 (2)	C2'—C3'—H3E	109.00
C3'—C2'—C6	111.4 (2)	H3C—C3'—H3D	109.00
C1'—C2'—C3'	110.2 (2)	H3C—C3'—H3E	109.00
C1''—C2''—C2	112.6 (2)	H3D—C3'—H3E	109.00
C1''—C2''—C3''	110.4 (2)	C2''—C3''—H3F	109.00
O1''—C2''—C1''	107.2 (3)	C2''—C3''—H3G	109.00
O1''—C2''—C2	107.3 (2)	C2''—C3''—H3H	109.00
O1''—C2''—C3''	108.1 (2)	H3F—C3''—H3G	110.00
C2—C2''—C3''	110.9 (3)	H3F—C3''—H3H	110.00
C2—C3—C4	110.1 (3)	H3G—C3''—H3H	110.00
C3—C4—C5	110.9 (2)	C3—C4—H4A	109.00
C4—C5—C6	110.8 (3)	C3—C4—H4B	109.00
C2'—C6—C5	114.3 (2)	C5—C4—H4A	109.00
N1—C6—C2'	109.8 (2)	C5—C4—H4B	110.00
N1—C6—C5	107.8 (2)	H4A—C4—H4B	108.00
C2'—C1'—H1L	110.00	C4—C5—H5A	110.00
C2'—C1'—H1M	110.00	C4—C5—H5B	110.00
C2'—C1'—H1N	109.00	C6—C5—H5A	109.00
H1L—C1'—H1M	110.00	C6—C5—H5B	109.00
H1L—C1'—H1N	109.00	H5A—C5—H5B	108.00
H1M—C1'—H1N	109.00	N1—C6—H6	108.00
C2''—C1''—H1O	110.00	C2'—C6—H6	108.00
C2''—C1''—H1P	109.00	C5—C6—H6	108.00
C2''—C1''—H1Q	109.00		
			
C6—N1—C2—C2''	172.0 (2)	C2''—C2—C3—C4	179.1 (3)
C6—N1—C2—C3	−62.0 (3)	O1'—C2'—C6—N1	56.7 (3)
C2—N1—C6—C2'	−173.0 (2)	O1'—C2'—C6—C5	177.9 (2)
C2—N1—C6—C5	62.0 (3)	C1'—C2'—C6—N1	−61.9 (3)
N1—C2—C2''—O1''	−60.2 (3)	C1'—C2'—C6—C5	59.4 (3)
N1—C2—C2''—C1''	57.6 (3)	C3'—C2'—C6—N1	173.7 (2)
N1—C2—C2''—C3''	−178.1 (2)	C3'—C2'—C6—C5	−65.0 (3)
C3—C2—C2''—O1''	177.7 (2)	C2—C3—C4—C5	−54.9 (4)
C3—C2—C2''—C1''	−64.5 (3)	C3—C4—C5—C6	55.4 (4)
C3—C2—C2''—C3''	59.8 (3)	C4—C5—C6—N1	−56.7 (3)
N1—C2—C3—C4	56.2 (3)	C4—C5—C6—C2'	−179.0 (2)

### Hydrogen-bond geometry (Å, º)

**Table:**

*D*—H···*A*	*D*—H	H···*A*	*D*···*A*	*D*—H···*A*
N1—H1···O1′	0.84 (3)	2.38 (3)	2.792 (3)	111 (2)
N1—H1···O1′′	0.84 (3)	2.43 (3)	2.814 (3)	109 (2)
O1′—H1*A*···O1′′^i^	0.82	1.99	2.805 (3)	169
O1′—H1*B*···O1′^ii^	0.83	1.99	2.807 (4)	167
O1′′—H1*C*···O1′^i^	0.82	2.00	2.805 (3)	171
O1′′—H1*D*···O1′′^iii^	0.83	2.03	2.762 (5)	148

Symmetry codes: (i) *x*, −*y*+1/4, −*z*+1/4; (ii) −*x*+5/4, −*y*+1/4, *z*; (iii) −*x*+5/4, *y*, −*z*+1/4.
